# Is Camphor the Genus Epidemicus of the Present Cholera Invasion?

**Published:** 1893-05

**Authors:** G. R. Ball

**Affiliations:** Boston, Mass.


### IS CAMPHOR THE GENIUS EPIDEMICUS OF THE PRESENT CHOLERA INVASION?

G. R. Ball, M. D., Boston, Mass.

September 17th, I prepared quite a quantity of Spts. of Camphor, making a saturated solution, and was exposed to the strong fumes for about two hours.

September 18th, soon after rising, was taken with very severe cramps through abdomen, followed by a copious evacuation, markedly exhausting. Two hours later a second evacuation, not as copious but much more exhausting. I now took a single dose of Camphor$^{dm}$ (Swan). Rested quietly for an hour, keeping well covered and taking nothing into my stomach but a few spoonfuls of ice-water. Had a very comfortable afternoon and night, there being no more evacuations. September 19th in usual good health, excepting a slight weakness. I may add, I had eaten nothing which could have caused the attack.

Might not this serve to confirm Dr. Swan's hypothesis that each crude drug is completely antidoted by its own high potency?

